# China and the Global Uranium Market: Prospects for Peaceful Coexistence

**DOI:** 10.1155/2013/672060

**Published:** 2013-03-31

**Authors:** Pascale Massot, Zhan-Ming Chen

**Affiliations:** ^1^Department of Political Science, The University of British Columbia, Vancouver, BC, Canada V6T 1Z1; ^2^School of Economics, Renmin University of China, Beijing 100872, China

## Abstract

China's recent reemergence has resulted in a significant increase in the global demand of commodities and is already having major impacts on the dynamics of global commodity markets. In the case of the global uranium market, we stand at the very beginning of a period of change. However, interesting trends are already emerging. Whereas China has had many policy reversals, and some difficulties in taking control of its procurement strategy in other commodity markets, it is seemingly more successful in managing its uranium procurement strategy. Why? The argument presented here is that a mixture of domestic and international level variables has allowed China more room for maneuver in fulfilling its uranium procurement strategy. On the domestic level, a centralized industry, and, on the international level, a geographically dispersed and uncoordinated market have allowed China to forge ahead with an ambitious civilian nuclear power plan and triple its total uranium imports, all within the span of a few years. Many challenges remain, not the least that of negative public opinion, which has surged since the Fukushima disaster in 2011. Nevertheless, should uranium demand continue to grow, this paper will consider the potential for continued peaceful coexistence among uranium market participants worldwide.

## 1. Introduction

China's fast development has resulted in a significant increase in the global demand of almost all commodities. This increased demand has already had major impacts on the dynamics of certain global commodity markets [1–6].

In the case of the global uranium market, we stand at the very beginning of a period of change. China's uranium demand has only started to increase significantly in the past 10 years. Moreover, if many analysts are arguing that Chinese demand for certain commodities may plateau or even fall in the near future, the situation is clearly different in the case of uranium. China has 16 operating nuclear reactors as of mid 2012, but it has 27 reactors under construction and plans to build at least another 50 in the coming years [7]. 

Another reason why China's interaction with the global uranium market is interesting is the room for manoeuver that China was able to carve for itself in this global market. Whereas China has had many policy reversals, and some difficulties in taking control of its procurement strategy in the global market of iron ore, for instance, it is having a better time managing its uranium procurement strategy. Why is China seemingly more successful in its entry on the global uranium market? Is this emerging trend likely to persist? Considering the fact that uranium demand is projected to go up substantially in the coming years, what is the potential for continued peaceful coexistence and cooperation among uranium market participants worldwide? This paper seeks to answer these questions in turn.

In sum, the argument is made that specific components of the structure of the international uranium market allow China more room for maneuver than in many other commodity markets and are influencing the way in which China chooses to engage with it. The global structure of the uranium market is relatively dispersed geographically but, more importantly, exhibits a lack of coordination among the existing market stakeholders; it has also suffered from underinvestment and lack of interest for decades leading to the early 2000s and thus was ripe for welcoming increased investment and involvement by an emerging actor such as China. On the domestic side of things, the importance given to China's civilian nuclear program by the government and the concentration of decision-making among few actors have allowed the country to manage existing challenges and effectively advance its overseas procurement strategy.

In terms of prospects for future peaceful coexistence, they can be realized if the recent international cooperation initiatives, which encourage transparency and collaboration, can create a virtuous circle. In an unexpected way, nuclear safety and security concerns, which lead to international cooperation mechanisms in the first place, beyond enhancing nuclear safety in China, could provide the impetus for more cooperation internationally. These international efforts have also showcased China as a country ready to rise to the occasion and be a responsible player, as a member of the International Atomic Energy Agency for instance.

After a short review of the history of the uranium market since its emergence, the consequences of the Fukushima nuclear accident and the global structure of the uranium market will be discussed. Thereafter, China's current growing resource needs as well as the domestic and international challenges it faces will be reviewed. This will be followed by an assessment of the current Chinese uranium procurement strategy. In conclusion, an analysis of China's participation in recent cooperation initiatives bilaterally and internationally will be made. 

## 2. A Short History of the Uranium Market

First of all, “There are at least four markets in the front-end of the nuclear fuel cycle that must be reviewed to determine assurance of supply: (1) uranium mining and milling, (2) uranium conversion, (3) uranium enrichment, and (4) nuclear fuel fabrication.” [8]. See also Conde and Kallis [9]. This paper will concentrate mainly on the first step, uranium mining. 

### 2.1. Emergence—Atoms for Peace—Cartel Period

The uranium industry emerged in the late 1940s early 1950s, mainly for military purposes [10]. In 1953, the race for dominance in the area of civilian nuclear power was set in motion by the US Atoms for Peace program. 

In the 1960s, the American Energy Agency banned the use of foreign uranium in its domestic reactors and aggressively cut prices of its own uranium exports. This was a period of oversupply in the rest of the world. The Canadian government decided to support its domestic uranium industry while stockpiling its inevitable surplus of uranium production.

It was then that Canada and other major uranium producers of the world (Australia, France, South Africa, and Rio Tinto Zinc Ltd.), in the absence of the US, sought to mitigate the impacts of the American policy and resorted to the covert manipulation of the world market [11]. 

In June 1972, the secret international uranium cartel was formally established (arrangements included price fixing, bid rigging, and market sharing). The cartel was referred to as the Société d'Études de Recherches d'Uranium (SERU).

Westinghouse filed an antitrust action against the cartel members, including a Canadian company, in 1976, and the cartel subsequently dismantled. We have not been able to find evidence that uranium producers have continued to coordinate their operations since then. 

### 2.2. Oversupply and Fall in Prices

During the 1980s and 1990s, for a combination of reasons, including the end of the Cold War (and thus the increased availability of secondary sources of uranium), a lull in the construction of new nuclear power plants worldwide (because of the consequences of the Three Mile Island and Chernobyl disasters and a reduction in expected growth of electricity demand) resulted in a fall of the uranium spot price, and thus of mining production.

Indeed, from the early 1980s until 2001, uranium prices trended downward and remained between USD $7 and USD $10 a pound.

### 2.3. Price Bubble

Beginning in 2001, the price of uranium began to rebound from historic lows and continued to rise through 2007. The real bubble occurred during the year 2007, triggered by shrinking weapons stockpiles (and thus the decreased availability of secondary sources), a flood at the Cigar Lake Mine in Canada, expected undersupply due to a slew of reactors coming online, compounded by the relatively recent news of an extensive nuclear program expansion in China, as well as speculative pressures. 

**Figure 1:**
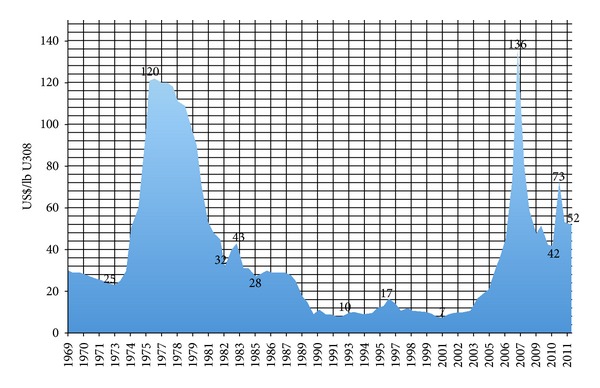
Uranium spot prices 1969–2012. Source: (1969–1989, NUEXCO Exchange Value, UX Consulting, constant 2007$; 1989–present, CAMECO, current$).

As the uranium price shot to historical heights of USD $136 a pound (see Figure 1), the extent of the twenty previous years of underinvestment in uranium production became all the more obvious.

### 2.4. Impact of Fukushima Crisis

The 9.0 magnitude earthquake in Japan was a uniquely severe natural disaster. So severe that the safety standards in place at the Fukushima power plant did not allow for such a magnitude. This was compounded by the impact of the tsunami. 

The international consequences of the Fukushima disaster were substantial, first and foremost in Japan. As of August 2012, only 2 out of the 54 Japanese reactors are operating again [12]. The government's basic stance on dealing with the power shortage has so far been to beef up its efforts to reduce power consumption and increase thermal power generation. But the combination of a fall in energy output and increased disruptions of supply is likely to have a lasting negative impact on growth, as argue Weinstein and Schnell [13]. Indeed, as Japanese communities continue to oppose the reopening of nuclear reactors, the effect of the earthquake has translated in a sustained drop in industrial production. The Japanese Minister of Industry (Yukio Edano) was quoted as saying that it is “necessary to restart nuclear reactors to avoid power shortages, provided that it can be done safely and with the agreement of local residents” [14].

The civilian nuclear industry seems poised to draw the lessons from this event, but it can be expected that the improvement in nuclear safety measures as well as the implementation of more efficient emergency procedures should have an inflationary impact on overall costs, just as it was the case after previous major accidents [15]. Such expectations, as well as increased public opposition to nuclear power, resulted in a drop of uranium prices worldwide. Between February 2011 and August 2011, the spot indicator fell by around 30% from a high of USD $72.63 a pound to US $49.13 a pound. 

In China, “five days after the earthquake and tsunami, the State Council suspended approval of new nuclear projects and started conducting comprehensive safety inspections of all nuclear projects—those in operation as well as those under construction. It also decided to halt four approved projects due to start construction in 2011” [16]. Chinese companies have responded to the changing global context by attempting to renegotiate outstanding bids and positions. In early May 2011, CGNPC withdrew its $1.24 billion bid to acquire a controlling interest in Kalahari Minerals PLC, which is developing a uranium mine in Namibia and then reopened the talks in the fall of 2011. A deal was finally struck again in February 2012 for close to $1 billion. 

### 2.5. Current Prospects

In the end, however, many analysts argue that the impact of the Fukushima accident on uranium prices will be short-lived, since the projected drop in demand will most likely be more than compensated by growth in emerging countries. Indeed, it is expected that Asia will account for most of the growth in new nuclear reactors, of which 40 percent will come from China [17].

“Globally, (the CEO of CAMECO Tim) Gitzel said he expected uranium demand to grow about 3 percent a year in the “next few years.” The “psychological” impact of the Fukushima nuclear accident in Japan will be in the “short-to-medium-term,” he said [18]. 

Current analysts actually forecast a growing deficit in uranium supplies starting from now (shortfalls have been driven by either lower forecast prices compared to 2007, problems with existing operations or delays in new mine production). For instance, the Royal Bank of Canada Capital Markets argues that uranium price indicators are down from the 2007 bubble, but still up from the 2008 low, and foresees uranium demand growing by an average of 4% per year during the next 20 years. They project a price of $80 a pound for 2013 [19].

While the fact that Germany decided to phase out its civilian nuclear program by 2022 made the news following the Fukushima disaster, talks of a nuclear phase out of nuclear energy for commercial power generation purposes had been on the agenda in Germany since at least 2002. German uranium demand represents anywhere between 2300 [20] and 3800 tons annually [21], or less than 5% of global demand. All in all, the construction of reactors in China is expected to outweigh the decommissioning of plants in Germany, Japan, Belgium, Italy, and Switzerland.

Bullish price predictions are also supported by the fact that we are currently entering a key transition period for the uranium market. Current global uranium production meets around 75% of global demand, the rest being met by stockpiles (released from military sources), inventories of which are rapidly decreasing. Indeed, the US-Russian Highly Enriched Uranium (HEU) Purchase Agreement, which converts surplus HEU from Russian nuclear weapons into fuel for US commercial power reactors, is expiring in 2013 [19, 22]. 

This “Megatons to Megawatts Program is a unique, commercially financed government-industry partnership in which bomb-grade uranium from dismantled Russian nuclear warheads is being recycled into low enriched uranium (LEU) used to produce fuel for American nuclear power plants. USEC, as executive agent for the U.S. government, and Techsnabexport (TENEX), acting for the Russian government, implement[ed] this 20-year, $8 billion program. (…) In years past, up to 10 percent of the electricity produced in the United States has been generated by fuel fabricated using LEU from the Megatons to Megawatts program.” [23].

The Director General of Rosatom says the contract will not be renewed in 2013. This will cause a gap of around 20,000 tons of uranium. Only around half of this gap will be filled through a new supply agreement that was signed between Russia's Techsnabexport (TENEX) and the United States Enrichment Corporation. Whereas the Megatons to Megawatts program was special in that it converted Russian nuclear weapon material, the new 2013–2022 supply agreement will provide the US with commercially enriched uranium from Russia [24]. Adding to the glut, many large uranium mines are currently close to depletion.

The uranium spot market reflected a persistent buyers' market over the 15-year period of 1980 to 1994, and again between 1998 and 2003 [25]. The situation is reversed to a significant degree now. 

### 2.6. Uranium Spot Market

Due to its special history, uranium is not widely traded on an organized commodity exchange, such as the London Metal Exchange. Spot prices are responsible for no more than 15% of global trades. For instance, during 2008, about 48 million pounds of U_3_O_8_ changed hands in the spot auctions [26]. This is a relatively small quantity compared to the 250 million pounds of uranium contracted by world utilities in 2005. 

It is true that the long-term uranium market and spot prices have a tendency to move together, because of “market-related” price mechanisms built in long-term contracts, and the fact that long-term contracts have quantity flexibilities built in them [25]. However, as Gene Clark, formerly with the US Department of Energy, said in an interview for UraniumSeek, a lack of liquidity and sophisticated market mechanisms remain features of the global uranium market [25].

It remains to be seen whether the emergence of trading mechanisms that we have seen in other markets (coal and iron ore come to mind) will extend to the uranium market. In 2007, the New York Mercantile Exchange signed a 10-year agreement with UX Consulting Company, to introduce U_3_O_8_ swap futures on CME and NYMEX platforms [27].

**Figure 2:**
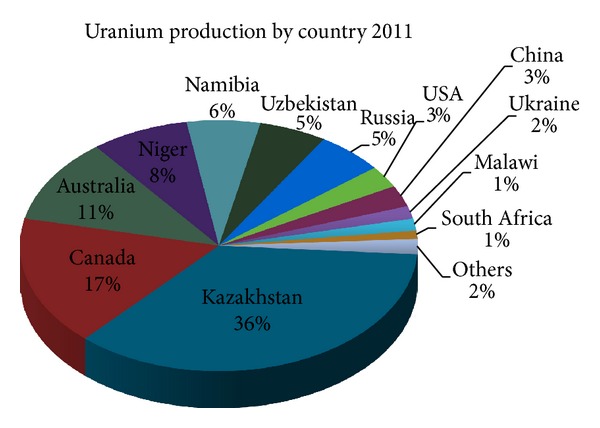
Uranium production by country in 2011. Source: Euratom Supply Agency, World Nuclear Association.

As of 2006, the 6 biggest uranium mining companies occupied 77% of the global market (Areva-17%, Cameco-16%, Rio Tinto-16%, Kazatoprom-13%, ARMZ/Rosatom-9%, and BHP Billiton-6%) [28]. In 2011, 4 countries contributed 72% of global uranium production (see Figure 2). Therefore, on the one hand, this indicates that the global uranium market is relatively concentrated. 

However, two characteristics of this global market deserve further attention. First, the uranium market has neither a liquid spot market nor a producer's organization (its 1970s cartel having been disbanded), it is relatively fragmented geographically, and the largest producer of uranium really only emerged within the last decade. So on the other hand, the global uranium market is a thinly institutionalized market. Second, while the purpose of this paper is not to delve in the technical details of nuclear fuel production, suffice it to say that uranium is an unusual commodity in that it necessitates highly capital-intensive transformations (enrichment) prior to being used as fuel, a step that can be (and often is) conducted in a different country than the country of uranium ore extraction (see Table 1), and a step that is closely related to security issues. China does possess enrichment capabilities, so it mainly only needs uranium ore, but this still complicates the picture further. 

Perhaps because of historical security concerns, and the absence of a widely used spot market, the global uranium market also remains a market where state-to-state relations continue to play an important role. Some recent major developments in Chinese procurement contracts were the result of state-to-state negotiations, including, recently, with the Canadian Government (more details below). The fact that China has ongoing long-term relationships with its close neighbours in Central Asia, such as with Kazakhstan and Uzbekistan, has also played a part in its ability to sign procurement agreements with them. 

## 3. China's Projected Uranium Demand and Procurement Strategy

### 3.1. Current Needs and Supply-Demand Gap

Whereas a key dimension of China's energy security aims [29] has been to rely as much as possible on domestic production [30], more recently, an emphasis on rapid expansion of electricity production, diversification of the energy mix, as well as environmental protection has contributed to the emergence of China as a civilian nuclear power [31–33]. In 1991, China connected its first nuclear reactor to the electricity grid [34]. In 2002, only 2 nuclear reactors had been built in China, but the country was already firmly looking ahead towards a future where nuclear energy would produce between 40 and 80 GW [31]. “During the 10th 5 Year Plan (2001–2005) period, the key part of China's energy policy [was] to ‘guarantee energy security, optimize energy mix, improve energy efficiency, protect ecological environment, continue to open up wider, and speed up the development of the west regions' [sic].” [31]. 

Five days after the earthquake and tsunami, the State Council suspended approval of new nuclear projects and started conducting comprehensive safety inspections of all nuclear projects. However, if there were any doubts as to whether China would continue to go ahead with its ambitious civilian nuclear program following the Fukushima disaster and the year-long safety review, these doubts were dispelled early this year. In his speech for the Nuclear Security Summit in Seoul in March 2012, President Hu Jintao underlined the “irreplaceable role of nuclear energy in ensuring energy security and climate change” [35]. This was a signal that echoed Wen Jiabao's comments made a couple of months earlier in Abu Dhabi, where he said that “Nuclear power is a safe, reliable, mature technology providing clean energy. The safe and efficient development of nuclear power is the solution to future energy supply strategy.” [36].

Then, “the former head of the NEA [National Energy administration] said that full-scale construction of nuclear plants would resume in March 2012” [37]. This confirmed that China is going ahead with its extensive expansion of civilian nuclear power plant program, albeit potentially at a slower rate: the target set by the National Development and Reform Commission (NDRC) in 2007 to have 40 GWe (Gigawatt-electric) online by 2020 [38] was upgraded to 70–80 GWe in 2010 and revised to 60–70 GWe in the aftermath of the Fukushima accident [37]. Currently at least 27 reactors are under construction [36] (or 26, according to the World Nuclear Association, see Table 2) and 50 more are planned according to the China Nuclear Energy Association [7]. 

As a consequence, whereas China's share of the market is still relatively low, planned construction of nuclear power plants is ambitious and China may be the first ranked importer of uranium globally by 2020. Qian Zhimin (China National Energy Administration) argued that by 2020, nuclear power could be contributing 7%-8% of China's energy needs, a higher rate than the official government target of 5% [39].

But such an ambitious civilian nuclear program coupled with very limited Chinese uranium reserves will only exacerbate China's import dependency ratio in this area, which is already high. As emphasized by Xiao Xinjian in China Energy, China has no choice but to develop a strong foreign procurement strategy in light of the country's poor uranium resources [40]. 

China's known uranium resources are insufficient: China total possesses at most 1 percent of the world's known recoverable uranium resources or about 68,000 tons [41]. The country's uranium output in 2011 was only 1500 tons (about 3% of global production, see Table 3), while its annual consumption had been at around 4,500 tons up to 2012 (or about 2% of global consumption) [42]. Its output is expected to eventually rise to 2,500 tons a year according to UX Consulting [7], but the quality of China's uranium resources is poor [43]. Clearly though, additional supplies are required and increasing dependence on imports is unavoidable in light of current development plans. China's imports may rise to about 17% of global consumption, or about 40,000 tons every year by 2020 [44].

Presumably in anticipation of a rapid increase in its uranium demand, China is already importing more uranium than it needs in a given year. For instance, China Daily indicated that China imported 17,135 tons of uranium in 2010, and 16,126 tons in 2011, according to the Chinese Customs [7], more than triple the amount in 2009.

### 3.2. Domestic Challenges

At the Chinese domestic level, many institutions with widely varying responsibilities are charged with ensuring nuclear safety, but these institutions all have different mandates and pursue different objectives. Similarly, at the regulatory level, a set of independent agencies exists, and this can create overlap issues. In addition, there remains work to be done on the level of the human capital needed to ensure the safety of the civilian nuclear program.

Hu Jintao emphasized this in his March 26th speech in Seoul: “we should establish and improve nuclear safety legal and regulatory system, strengthen the building of a nuclear emergency team, increase investment in research, staff training, and provide an institutional guarantee for strengthening nuclear safety, in response to the emergency mechanism to protect, and provide technical support to raise the level of nuclear safety human resources and support to enhance nuclear safety” [35].

These efforts, while enhanced since the Fukushima disaster, were built on earlier commitments to nuclear safety. In 2006 Kadak argued that

*“China has developed top-level nuclear safety regulations on site location, safety in design, operations, and quality assurance. They annually set up inspection plans for each power station which focus on key areas of the regulations to assure compliance. They also have special inspections and reviews based on events that may occur at the plant. While the organizational framework is quite similar to the U.S. system, the intrusiveness of the regulator in day-to-day operations is not. The onsite inspectors follow the overall plans for inspections but are not as involved in day-to-day oversight of normal operations and outages. (…) Chinese regulatory personnel do perform inspections and oversee major activities during outages, however.*

*(…) The Chinese government has made it quite clear that they will not tolerate injuries or radiation release. Given the power of the Chinese government, this clarity in expectations of the regulator and the government makes operational decisions of the plant very safety-focused. The challenge is for management to reinforce these expectations to all plant employees and contractors to be sure that no unsafe conditions exist [32].*

In recent years—and this trend has been compounded by the Fukushima disaster—we have seen the rise of another domestic challenge for the government in China, that of increased public awareness of the risks involved with nuclear power. This increased awareness, coupled with better access to information and means to express opposition via the Internet, has produced much activity online. A case in point is the opposition to the Gaozuang power plant in Nanyang, Henan province [45].

Despite domestic challenges, China has been able up to now to forge ahead with its national nuclear energy plan (as we have seen, albeit at a somewhat slower pace following the Fukushima disaster). 

This has much to do with the fact that the Chinese nuclear industry is centralized domestically. Indeed, the civilian nuclear industry and the uranium mining industry are overwhelmingly controlled by two state-owned enterprises—China National Nuclear Corporation and China Guangdong Nuclear Corporation—that report directly to the State Council [41]. The structure of the domestic uranium market might therefore have afforded the Chinese government a stronger hand in fulfilling its policy goals (contrary to other more fragmented markets, such as iron ore, where the government has struggled). This may have allowed China more room for maneuver to concentrate on fulfilling its procurement strategy abroad. 

### 3.3. International Challenges

Chinese companies also face challenges in fulfilling their uranium procurement needs internationally. This point was emphasized by Xie Qingxia, Hua-ming, and Wu Ping in their 2011 China Mining Magazine article [46]. The authors say that challenges lie in the form of a dearth of experience in the management of global uranium extraction companies, lack of knowledge in the legislation and domestic policies of foreign countries, the presence of political risks in host countries and the fact that China is a late player in the game. 

In terms of political risks, besides possible diplomatic tensions, China must also manage potential issues to do with corruption and political instability with the source countries, including in Central Asia and Africa.

Further, because of the predicted excess of global demand over global supply in coming years, the Central Asian uranium market will remain very competitive. China is competing for access with Russia and India in Kazakhstan and Mongolia, while South Korea and Japan also buy significant amounts of uranium there and Iran is looking to raise its import as well. India is competing with China in Namibia and Niger as well. Thus, China is not operating alone in markets it is arguably the most comfortable in. Therefore, although China has been trying to diversify its supply sources and shown caution not to overbid for resource acquisition, in the end, strong competition has compelled it to make use of economic and diplomatic tools to gain an edge. Indeed, “the Chinese have shown they will often pay above market prices for those mines, companies and other assets that are genuinely rich in natural resources” [47]. In many ways, uranium market pressures continue to be resolved within a state-to-state framework. 

Another type of political risk is the risk of falling afoul of domestic public opinion in source countries. This risk exists in developed source countries. For instance, the Australian population reticence towards nuclear power domestically and abroad has had an impact on the country's export capacity. Since the 1970s, the country has seen ongoing debates between the uranium mining and nuclear industry and environmentalists and indigenous land rights activists. Australia also suffers from other usual obstacles to uranium mining, including in the form of shortage of labour and infrastructure [48]. But it is Australia's 1984 “Three Mines Policy” [9] and subsequent “No New Mines” policy that really limited the scope of uranium mining in the country, until a recent, and timid, loosening. 

Social opposition and resulting stricter environmental regulations are thus partly responsible for the transfer of uranium mining away from developed countries in recent years. 

However, public opinion risks do not come exclusively from developed economies. Indeed, when it comes to planning investments in developing countries, Chinese companies need to successfully manage the public perception of their actions as well. Following the Fukushima incident, public opposition has risen in Kazakhstan, already exacerbated by years of environmental and safety mismanagement during the Soviet period. “The Chinese have apparently sought to decrease this risk by partnering with the state-owned Kazatomprom in exchange for equity in domestic facilities.” [47].

This situation is not unlike that found in other commodity markets. Indeed, China was not a major stakeholder when global commodity market institutions emerged during the second half of the twentieth century and has found itself a newcomer in many markets as it rose to prominence in the past two decades. But in the case of uranium, the fact that the market does not have a producer's cartel, and was in need of investment and expansion as China entered the market, has given China more room to maneuver. 

### 3.4. Procurement Strategy

China has been basing its uranium procurement strategy on the national “Two markets, Two resources” policy, coined in the 1990s, which works to develop both domestic supply sources (this includes developing advanced nuclear power systems and/or alternative nuclear power methods to save fuel) and international supply sources (through foreign acquisition, investment as well as long-term contracts). “By 2020, 1/3 of China's supply of natural uranium will come from domestic uranium production, 1/3 from direct procurement from foreign suppliers, and 1/3 from the overseas holdings of uranium production.” [46]. Such a diversified strategy can spread the risk associated with a high import dependency ratio. 

Therefore, on the one hand, China is increasing its uranium production domestically (see Table 3). On the other hand, as part of the “Going Out” strategy (which fits under the “Two Markets, Two Resources” policy), China is taking steps to acquire uranium resources internationally. It has equity in Niger and Kazakhstan mines, is investigating Uzbekistan, Mongolia, Namibia, Algeria, and Zimbabwe, and other sources are progressively being added. A bilateral safeguards agreement will also allow imports from Australia, and more recently, Canada.

Beijing is also using creative ways to engineer procurement contracts. As mentioned above, in November 2006, two Chinese firms have established a joint venture with Kazakhstan's state-owned Kazatomprom in a uranium mining project (49 percent stake), in exchange for stakes in either Chinese nuclear power plants or fuel reprocessing facilities for Kazatomprom [49]. China has also “provided interest-free soft loans to the governments of Uzbekistan, Niger and other uranium-rich countries (World Nuclear News, June 10, 2010; November 4, 2008; Reuters, April 24, 2010)” [47].

We stand at the beginning of China's engagement with the global uranium market. China has thus an opportunity to learn from past experiences acquired while dealing with high import-dependency ratios in other types of commodities. Up to now, China has developed a multi-pronged strategy of engagement in the uranium market, that has allowed it to triple its total uranium imports, and forge ahead with the world's most ambitious civilian nuclear power development plan, all within the span of a few years.

## 4. Potential for International Cooperation

### 4.1. A Case in Point: Bilateral Cooperation between China and Canada

*Uranium Ore.* Uranium trade relations between Canada and China show that there remains a large state-to-state angle to this trade. Among the largest uranium producers, Canada has the highest-quality uranium. Indeed, “only Canada has a significant amount of ore above 1 percent—up to about 20 percent of the country's total reserves. In Australia, on the other hand, some 90 percent of uranium has a grade of less than 0.06 percent. Much of Kazakhstan's ore is less than 0.1 percent” [48]. While the Fukushima accident reduced demand for new nuclear power plants in the near-term, CAMECO has said it is sticking to its target to double uranium production to 40 million pounds by 2018. Canada's uranium production is projected to increase at an average rate of 9 per cent a year to 15 300 tonnes in 2016.

During the Canadian Prime Minister's visit to China in February 2012, a protocol amending Canada's nuclear cooperation agreement with China to allow the export of uranium concentrate was announced. This was big news both for China's energy security and potential to diversify supply sources, and Canada, which can now export uranium directly to China. These sales were banned up until now. In 1976, Canada barred exports of uranium and nuclear reactors to countries that had not signed the Non-Proliferation Treaty (China signed in 1992). A 1994 agreement allowed the sale of reactors, but until last year's amendment to that pact, Canada hadn't yet relaxed its restriction on selling nuclear fuel to China. So until last year, Cameco Corp. had to ship the uranium that it was selling to China from other countries, such as Namibia and Kazakhstan.

In November 2011, Cameco signed an agreement with China Guangdong Nuclear Power Holding to import 29,000 tons of the mineral through 2025. Canada's Cameco has also contracted to sell 23,000 tons of uranium concentrate trough 2020 to China Nuclear Energy Industry Corporation (directly owned by China National Nuclear Corporation) [44].

Following this, Saskatchewan Minister of Energy Bill Boyd signed a Memorandum of Understanding (MOU) in February 2012, on scientific and technical research co-operation on uranium geology with the Beijing Research Institute of Uranium Geology, a research establishment of the China National Nuclear Corporation. 

Cameco Corp. Chief Executive Officer Tim Gitzel said “China is becoming the leader of the world” for nuclear energy [50]. Gitzel said Cameco continues to discuss partnerships with Chinese companies on the possibility of jointly developing uranium projects in China, Canada, and other countries.

### 4.2. Technology

This relationship extends to technical levels of cooperation as well. Indeed, technological cooperation in civilian nuclear technology has occurred between Canada and China in recent years. Indeed, in 2009, the capacity of CANDU's heavy water reactors to reuse spent fuel recycled from other light water reactors was explicitly recognized by a panel appointed by the China National Nuclear Corporation. It “cited the design's ‘enhanced safety and good economics' as reasons it could be deployed in China in the near term” [51] and recommended that two units be built. 

China is also pursuing reprocessing capabilities in partnership with Canadian CANDU designer Atomic Energy of Canada. China has built two CANDU reactors (and is considering building additional units), which are utilizing reprocessed fuel from its nine light water reactors. CANDU reactors can also run on thorium fuel, and China has been working on developing a thorium fuel cycle with its Canadian partners. Thorium is more abundant in China, cheaper to mine, produces less waste and, if successful, will enhance Chinese energy security. 

As the case of Canada-China uranium and nuclear relationship shows, the uranium trade is nested within a thicker web of state-to-state relations that include safety and technological exchange issues. As will be developed further below, this embeddedness may have a positive impact on the nature of China's participation in the global uranium market [52, 53].

### 4.3. Cooperation on the Enrichment Level

Uranium being only the first step towards the development of nuclear fuel, cooperation initiatives regarding enriched fuel also play a role in the broader civilian nuclear fuel market. The World Nuclear Association lists operating uranium enrichment facilities in 11 countries, 6 with large capacity (France, the US, the UK, Germany, Russia and the Netherlands) and 5 of smaller capacity (China, Japan, Brazil, Pakistan, and Iran) [54].

There are multiple initiatives underway which seek to coordinate enrichment activities worldwide, and the International Atomic Energy Agency (IAEA) actively encourages these “Multilateral Nuclear Approaches” [54]. These programs contribute to enhancing the global tracking of enriched fuel, and thus fulfill nonproliferation goals, while facilitating the coordination of the global market for enriched fuel. A case in point is the Eurodif enrichment center in France, jointly owned by five countries (France, Belgium, Italy, Spain, and Iran), operating under the IAEA oversight, and giving the participants some controlled access to the final product without sharing any technology [54].

Similar initiatives are underway in Russia, where under the IAEA's supervision, an International Uranium Enrichment Centre is being created in Siberia, with Russian, Kazakh, Ukrainian, and Armenian equity. Other such projects are underway in the US, the UK, the Netherlands, and Germany. 

The IAEA is also calling for the establishment of international banks of enriched fuel. A deal was signed for Russia to make available 120 tons of nuclear fuel, and a similar arrangement was being discussed with Kazakhstan in 2010. Such initiatives, say former IAEA Director General Mohamed ElBaradei, “make sure that every country that is a bona fide user of nuclear energy, and that is fulfilling its nonproliferation obligations, is getting fuel” [48].

This only goes to show that uranium procurement is a part of broader civilian nuclear issues. China has displayed willingness to collaborate on security issues that pertain to civilian nuclear programs and nonproliferation, and additional participation in a global cooperation mechanism on uranium enrichment would further cement China's role as a constructive participant in such multilateral initiatives. 

### 4.4. Cooperation among Developing Countries

At the level of safety standards, China has indicated its willingness to play a leading role in fostering cooperation initiatives among developing countries. Indeed, the country has indicated that it is interested in providing nuclear safety assistance, and focuses on helping developing countries establish and improve their nuclear safety infrastructure, as well as improve nuclear safety and technical standards [35]. 

China has already been involved in multiple regional nuclear security training courses providing training to nearly 100 people from more than 10 countries in the Asia-Pacific region [35]. It also looks to help developing countries improve technical levels of nuclear safety.

This is yet another way in which the multidimensionality of the civilian nuclear industry manifests itself. China's engagement internationally in the realm of safety issues, and with developing countries (a setting where China has long played a role), may provide it with the experience and a platform to eventually broaden its engagement to other issues, for example, to issues of natural/enriched uranium procurement.

### 4.5. Impact of the Nuclear Security Summits

Enhanced cooperation on issues of nuclear security at an international level had already started with the Washington Nuclear Security Summit held in 2010, but the Fukushima accident brought back to the fore the need to enhance state capacity to cope with the unexpected and the need to address issues of nuclear safety. 

Indeed, the second Nuclear Security Summit held in Seoul in March 2012 broadened its agenda to include nuclear safety and proved to be an important step following the Fukushima accident and the need to discuss this issue in a multilateral setting. There is much to learn from the Japanese accident, and China has shown its willingness to learn from this experience. 

The summit is useful despite the fact that sovereignty concerns and economic and technological differences hinder the establishment of binding safety standards across the board. There are difficulties in harmonization of safety standards, but China has demonstrated a will to work in cooperation with other developed nations on this regard. It shows great confidence on China's behalf to fully engage developed countries in this sensitive issue.

“In July 2010 a 22-strong IAEA team from 15 countries carried out a two-week Integrated Regulatory Review Service mission to review of China's regulatory framework for nuclear safety. The IAEA made a number of recommendations but said that the review had provided ‘confidence in the effectiveness of the Chinese safety regulatory system and the future safety of the vast expanding nuclear industry.'” [37]. China has also “requested and hosted 12 Operational Safety Review Team (OSART) missions from IAEA teams to October 2011” [37].

In the past years, China has made substantial efforts in this regard. China has also made relatively noticeable efforts in reaching out regarding nuclear safety, as it has done with the US (implemented in 1998 and reinforced in 2005 by a Memorandum of Understanding that granted Westinghouse the contract to build four commercial nuclear reactors in China). China is part and parcel of the international community and its efforts to enhance nuclear security and safety globally [16].

A case in point, China is a fully fledged member of the International Atomic Energy Agency, committed to international nonproliferation efforts and cooperating on issues of civilian nuclear technology with France (Areva), Canada (Atomic Energy of Canada—CANDU), and the US (Westinghouse) among others, as well as participating in related international frameworks. 

China's energy needs are growing at such speeds that the parallel growth of its civilian nuclear program appears inevitable. But to do it, as President Hu Jintao emphasized in his Seoul speech, China needs to “face the risk of nuclear safety, to learn the lessons of the nuclear accident, and take effective measures to enhance security and reliability of nuclear energy, to promote the safety of nuclear energy, sustainable development” [35].

All in all, at the domestic level, a centralized industry, and at the international level, a geographically dispersed and uncoordinated market allowing some space for expansion have provided China with relatively comfortable conditions to roll out its procurement strategy abroad. But the fact that the uranium market remains one step away from nuclear security and safety issues, which have spawned a web of international cooperation initiatives, may foster a cooperative environment for uranium as well. 

It remains that whereas the structure of the global uranium market have allowed China to carve itself a place and roll out its procurement policy, the safety and security dimensions of the nuclear industry have provided China with the opportunity for a cooperative and confident engagement at a multilateral level. 

Many uncertainties remain, namely, the potential for a supply squeeze, delayed production due to mining accidents, and public opinion protests among others, so peaceful coexistence, or even cooperation, is not a foregone conclusion. Indeed, it remains to be seen whether a conceptual frontier will remain between natural uranium ore mining and enriched uranium production and other civilian nuclear issues. Further research could look at the likelihood of spilling effects. However, the particular conditions under which this market is evolving currently allow for such a possible development. 

## 5. Conclusion

The first signs of China's likely future impact on the global uranium industry have already been felt. Despite China's uranium requirements being less than half than that of the US, China still imported almost as much uranium as the US did in 2010, three times more than the amount it imported in 2009. This is despite the fact that the US has over 100 reactors in operation, against China's 15. 

Early signs also point to a reorganization of the global uranium market, with the emergence of new players such as Kazakhstan. This sudden increase and investment in Kazakhstan's uranium mining industry has contributed to the rise of this country to number one uranium producer in the world, whereas it was all but absent from global uranium trade in 2003 [19]. 

The answer as to why China has succeeded in establishing itself as a confident player pushing forward a multi-pronged international uranium procurement strategy has to do with both domestic and international variables. On the one hand, at the domestic level, the centralized structure of China's uranium procurement industry has allowed industry and government stakeholders to develop and implement a coherent strategy (contrary to the situation in the fragmented iron ore market for instance). On the other hand, at the international level, the absence of an exporters' cartel or oligopoly established before China's emergence as a large purchaser have enabled it to carve itself a place on the global market. 

On the top of that, recent international cooperation initiatives, which encourage transparency and collaboration, even if they are concentrating on nuclear safety and security issues, have provided China with the opportunity for a cooperative and confident engagement. These international efforts have showcased China as a country ready to rise to the occasion and be a responsible player, including as a fully-fledged member of the International Atomic Energy Agency. 

As the country builds confidence in dealing with these issues in an international setting, could it lead it to spearhead further initiatives, such as an international uranium demand management initiative, that could be managed through the Shanghai Cooperation Organization? China seeks to have a say in global market institutions, which for the most part have been created prior to its recent reemergence, that is commensurate to the share of the global demand that it now occupies. Demand management initiatives like this could be an area where there is room for China to innovate, perhaps in its own regional setting at first [55]. 

All in all, there is potential for peaceful coexistence—and even international cooperation—in the global uranium market, something towards which China can contribute meaningfully.

## Figures and Tables

**Table:** (a)

Top 5 exporters of uranium and thorium ores (2008–2011)	Trade value in USD	Top 5 exporters of natural uranium and its compounds (2008–2011)	Trade value in USD
Namibia	$2,803,841,482	Canada	$7,152,512,173
Niger	$1,549,622,598	USA	$2,578,682,476
Australia	$1,152,033,863	France	$2,568,788,240
Malawi	$243,107,642	Kazakhstan	$1,593,772,641
United Kingdom	$17,024,333	Russian Federation	$322,983,583
Total world	$5,777,745,980	Total world	$15,131,504,216

**Table:** (b)

Top 5 exporters of enriched uranium (2008–2011)	Trade value in USD
France	$10,755,771,990
USA	$5,493,937,151
Germany	$4,016,817,724
Netherlands	$3,631,753,994
China	$305,168,764
Total world	$24,623,102,401

Source: UN Comtrade.

**Table 2:** The 8 biggest civilian nuclear powers: planned reactors and uranium requirements in 2012.

Country	Reactors operable	Reactors under construction	Reactors planned and proposed within 15 years	Uranium requirements in 2012 (Tonnes U)
China	15	26	171	6 550
France	58	1	2	9 254
Japan	51	2	15	4 636
Russia	33	10	41	5 488
South Korea	23	3	6	3 967
Ukraine	15	0	13	2 348
United Kingdom	17	0	13	2 096
United States	104	1	30	19 724
World	435	62	489	67 990

Source: World Nuclear Association, April 2012.

**Table 3:** Natural uranium production in 2010, compared with 2009 (tons of uranium).

Country	Production 2011 (tU)	Production 2010 (tU)	Production 2009 (tU)	Share in 2011 (%)	Share in 2010 (%)	Share in 2009 (%)	Change 2011/2009 (%)
Kazakhstan	19 451	17 803	14 020	36%	33%	28%	39%
Canada	9 145	9 783	10 173	17%	18%	20%	−10%
Australia	5 983	5 900	7 982	15%	11%	16%	−25%
Niger	4 351	4 198	3 243	8%	8%	6%	34%
Namibia	3 258	4 496	4 626	6%	8%	9%	−3%
Uzbekistan	3 000	2 400	2 429	6%	4%	5%	24%
Russia	2 993	3 562	3 564	6%	7%	7%	−16%
USA	1 537	1 660	1 453	3%	3%	3%	6%
China	1 500	827	750	3%	2%	1%	100%
Ukraine	890	850	840	2%	2%	2%	6%
Malawi	846	670	104	2%	1%	0%	713%
South Africa	582	583	563	1%	1%	1%	3%
Others	1 074	931	1 025	2%	2%	2%	5%

Total	54 610	53 663	50 772				8%

Source: Euratom Supply Agency, World Nuclear Association.
